# Article Retraction Notice

**Published:** 2019-10

*The Journal of Tehran University Heart Center* hereby declares the retraction of the article entitled “A Rare Case of Cardiogenic Shock following Severe Multivessel Coronary Vasospasm”, which was already published in this journal (J Teh Univ Heart Ctr 2019;14:28-32, DOI: https://doi.org/10.18502/jthc.v14i1.653).

This article has been retracted at the request of its authors, who stated that notwithstanding the merits of their reported case, the complicated manifestations of the patient require further causal investigation via a more scrutinized approach.

